# Froth-Flotation Separation as an Alternative for the Treatment of Soil Enriched with Fluorine Derived from Mica

**DOI:** 10.3390/ijerph19031775

**Published:** 2022-02-04

**Authors:** Jeonghwan Cho, Moon Young Jung, Hwan Lee, Jinsung An

**Affiliations:** 1 Department of Environment Safety System Engineering, Semyung University, 65 Semyung-ro, Jecheon-si 27136, Korea; jcm20935@naver.com; 2 Department of Biological & Environmental Engineering, Semyung University, 65 Semyung-ro, Jecheon-si 27136, Korea; myjung@semyung.ac.kr; 3 SG Institute of Environment Science & Technology, 42, Soryong 1-gil, Gunsan-si 54012, Korea; hwanid@sgiest.re.kr

**Keywords:** fluorine-enriched soil, mica mineral, natural origin, froth-flotation separation, soil remediation

## Abstract

Fluorine (F) enrichment originating from natural sources is difficult to remove using chemical washing methods due to the large chemical-resistant residual fraction. This study evaluates the feasibility of using a froth-flotation separation method to remediate soil with a high F concentration caused by mica weathering, and it investigates the optimal conditions for this process, including pH of the slurry, collector dosage, and sample mechanical preparation strategy. The established optimum conditions are pH 3.5, 300 mg/kg collector dosage (tallow amine acetate), which can effectively separate quartz and mica, and a sieving-and-milling strategy that involves discarding particles of size < 0.05 mm, milling those in the range of 0.5–2.0 mm (until < approx. 0.3 mm), and mixing particles with sizes in the range of 0.05–0.5 mm. The target contamination level of 400 mg/kg for the test soil was not met after the first flotation separation process. However, after milling the residue of the first process and subjecting it to a second flotation separation process, the required contamination level was achieved. Consequently, the proposed froth-flotation separation process can be used as a successful alternative technique to remediate F-enriched soils from natural origin that have highly chemical-resistant forms.

## 1. Introduction

Fluorine (F) is the thirteenth most abundant element in the earth’s crust, with an average concentration distribution of 500–700 mg/kg [1,2]. Small quantities of F are added to some toothpastes for dental health, but ingesting large quantities can cause dental fluorosis, skeletal fluorosis, and osteoporosis [3,4,5]. The World Health Organization reported that the ingestion of F may cause cancers such as osteosarcomas and bone tumors [6].

The accumulation of F in soil is not only caused by human activities (e.g., phosphoric fertilizer use, coal use, aluminum and steel industry activities, and leakage accidents) [7,8,9], but also by the weathering of rocks containing F (natural origin) [10,11]. Fluorite (CaF_2_), mica (e.g., muscovite (KAl_2_(AlSi_3_O_10_)(F, OH)_2_) and biotite (K(Mg, Fe)_3_AlSi_3_O_10_(F, OH)_2_)), amphibole ((Na, K)_0–1_(Na, Zn, Li, Ca, Mn, Fe^2+^, Mg)_2_(Mg, Fe^2+^, Mn, Al, Fe^3+^, Ti, Zn, Cr)_5_(Si, Al, Ti)_8_O_22_(OH, F, Cl)_2_), apatite (Ca_5_(PO_4_)_3_(OH, F, Cl)), and cryolite (Na_3_AlF_6_) have been found to contain F [12,13,14,15]. The weathering of these minerals can lead to the accumulation of F in the soil and groundwater [16,17].

Typical methods for treating F-contaminated soil (i.e., removing F from soil) include soil washing and electrokinetic remediation [18,19,20,21,22,23,24]. Soil washing removes F by leaching it entrapped in, and/or adsorbed on, the soil particles using an acidic or basic washing solution. Moon et al. [21] conducted washing experiments on F-contaminated soil near chemical factories using various washing solutions (HCl, HNO_3_, H_2_SO_4_, NaOH, and C_4_H_6_O_6_). Among them, 3 M HCl exhibited the highest removal efficiency (approximately 97%). Meanwhile, Ahn et al. [18] reported that 1 M H_2_SO_4_ showed the highest removal efficiency (26.7%) in the treatment of F-contaminated soil. The form of F in the soil is believed to have caused the reduced removal efficiency compared to that reported by Moon et al. [21]. Ahn et al. [18] also evaluated the speciation of F in the soil using the sequential extraction method and reported that the residual F fraction increased from 80.5% before soil washing to 98.9% after soil washing. Considering that soil washing removes contaminants by extracting those adsorbed to the surface of soil particles using a washing reagent [25,26,27], the removal efficiency may decrease considerably if the residual fraction (i.e., highly chemical-resistant forms) of F is as high as that reported by Ahn et al. [18]. Electrokinetic remediation removes contaminants on the soil surface by detaching them through an electrolysis reaction that occurs near an electrode [20,22,23,24]. However, this method is only applicable to saturated soil with low permeability [20]. Noting that alkaline conditions facilitate detachment or dissolution of F, Kim et al. [20] treated F-contaminated soil using the electrokinetic method with NaOH as the anolyte and achieved up to 75.6% removal efficiency. Furthermore, they reported that this method can also be effectively applied to the removal of anionic contaminants, e.g., arsenate and chromate.

The target site of this study has been reported to exceed the Korean worrisome level of soil contamination (level 2 area: 400 mg/kg), owing to the accumulation of F in the soil through the weathering of mica, such as biotite, lepidolite, muscovite, and phlogopite [19]. The F-enriched soil of this site had a residual fraction of more than 99% [19]. The basic principle of both the soil washing and the electrokinetic methods is the removal of F by detaching them from soil particles using a suitable washing solution and anolyte. Therefore, it is difficult to use these methods to remove the F in soil derived from F-containing minerals because of its strong resistance to extracts. In fact, the applicability of chemical washing methods was evaluated under various washing conditions for the soils collected from the target site, and it was found that chemical washing was not feasible, as the maximum removal efficiency was only 6.2% [19]. Therefore, to remediate F-enriched soils of natural origin (i.e., soils enriched with F from mica weathering) to acceptable levels, the F-enriched mineral particles must be physically separated out.

This study assessed the applicability of the froth-flotation separation method, which is the process of physically separating minerals using differences in their wettability, to remove F in soil derived from mica minerals. It relies on the phenomenon of minerals with hydrophobic surface properties (owing to natural characteristics or added reagents) becoming more buoyant when air bubbles adhere to them [28]. The froth-flotation separation methods have been used for several decades in the field of mineral beneficiation, but the literature used for soil remediation (heavy metals, hydrophobic organic compounds, and crude oil-contaminated soils) has been reported since 2000 [25,26,29,30]. Nevertheless, to our knowledge, there is no previous research that has evaluated the feasibility and optimization of the froth-flotation separation method to remediate soil enriched and/or contaminated with F. This study aimed to evaluate the applicability of the froth-flotation separation method to soils with a high F concentration caused by the weathering of F-containing mica minerals and to determine the optimal conditions of processes to satisfy the Korean worrisome level of soil contamination (400 mg/kg) within a relatively short remediation period.

## 2. Materials and Methods

### 2.1. Sample Preparation and Characterization

Soil enriched with F that exceeds the Korean worrisome level of soil contamination (level 2 area: 400 mg/kg) was sampled at depths of approximately 1.5–2.5 m below the surface. Since it is a site where underground development is planned in the future, it is necessary to confirm the contamination characteristics and remediation efficiency of the soil in a relatively deep depth. The study site soil contains accumulated F derived from mica minerals [19]. After releasing the particles that had aggregated through wet disintegration, the gravel was separated using a 2-mm sieve (10 mesh). To determine the distribution of F concentration among the particle sizes of the soil sample, the specific particle size groups were separated using standard sieves (10, 35, 100, and 200 meshes) and a sieve vibrator (Analysette 3, FRITSCH, Idar-Oberstein, Germany). All experiments were conducted in triplicate. The F concentration in the soil was measured using the alkali fusion method [31]. After reacting 6 mL of 16 M NaOH and 0.5 g of dry soil sample at 600 °C for 30 min, the pH was adjusted to 8–9 for removing the aluminum and iron that can interfere with F analysis, and the F concentration was measured using a fluoride electrode (F001502, ISTEK, Seoul, Korea), with the aid of total ionic strength adjustment buffer (TISAB).

To determine the optimal pH conditions of the froth-flotation separation process for treatment of F-enriched soil, the pure minerals (biotite and muscovite) known to cause F accumulation in the soil of the study site were purchased from Geoscience Resources (30-1291: biotite, 30-1251: muscovite), and were ground and sieved to obtain a particle size of 0.075 to 0.5 mm. In order to check the separation efficiency of mica and quartz, the mineral that accounts for the largest proportion in the soil, pure quartz (30-1660: quartz) was also purchased and mixed with biotite. The X-ray diffraction (XRD) analysis results of the pure minerals used are shown in Figure S1 (Supplementary Materials).

### 2.2. Micro Flotation Tests

#### 2.2.1. Determination of Operational Parameters Using Pure Minerals

The optimal pH for the froth-flotation separation was determined for biotite, muscovite, and a mixed sample of biotite and quartz (i.e., to distinguish the two minerals by color with the naked eye) using a micro flotation separator (XFG50-100, Jiangxi Yunhao Mining and Metallurgy Equipment Co., Ltd., Ganzhou, China). Tap water (200 mL) was poured into a flotation separation cell, and 1 g of each pure mineral sample was added. While stirring the pulp (i.e., the mixed solution of tap water and pure mineral sample) at 1200 rpm, the pH was adjusted to 1.5, 2.5, 3.5, 4.5, 5.5, and 6.5 using concentrated H_2_SO_4_ (Daejung, Korea). To increase the hydrophobicity of the mineral surface, 1000 mg/kg-soil of tallow amine acetate (Armac-T, Akzo Nobel, Stenungsund, Sweden) as a collector was used. Additionally, one drop of aerofroth 65 (AF65, H(C_3_H_6_O)_6.5_OH, Cytec, Botlek, Netherlands) (i.e., approximately 50 mg/kg-soil) was added to make the target minerals rise above the water. After reacting for 3 min, 80 L/h of compressed air was injected for 5 min, and the floating foam including the target mineral was recovered.

The flotation yield of the pure mineral (biotite and muscovite) was calculated using Equation (1):

(1) $$Y_{f}=\frac{F}{R}\times 100$$

where *Y_f_* is the flotation yield (%), *R* is the weight of the raw sample (g), and *F* is the weight of the floating sample (g).

#### 2.2.2. Determination of Operational Parameters Using Mica Minerals Collected from the Sample

The mica minerals, which were presumed to be the cause of the F accumulation in the soil of the study site, were collected from gravels (≥2 mm) in the sample by hand-picking (Figure S2). After milling, the mica mineral was washed at a solid–liquid ratio of 1:1 (g:mL) using 1 M H_2_SO_4_, and the washing liquid was removed after approximately 1 min. The F concentration of the sample was analyzed using the alkali fusion method. If the F concentration in the mica minerals collected manually was higher than that in the soil sample (≤2 mm), the cause of the F accumulation in the soil could be attributed to the weathering of F-containing mica minerals. After performing the froth-flotation separation on the collected mica mineral sample using the optimal pH condition (i.e., pH 3.5) with the same concentrations of the collector (Armac-T = 1000 mg/kg-soil), and the frother (AF65 = 50 mg/kg-soil) and the same flow rate (80 L/h) and injection time (5 min) of the compressed air, the particle size of the floating product was measured using a particle size analyzer (LA-350, HORIBA, Kyoto, Japan) to determine the optimal particle size (i.e., sample milling conditions).

#### 2.2.3. Surface Characterization Using FT-IR

To demonstrate the reaction mechanism between the mica minerals and the collector (Armac-T) used in this study, Fourier transform infrared spectrometry (FT-IR, IRTracer-100, Shimadzu, Kyoto, Japan) was used. The Armac-T (10,000 mg/kg-sample) was injected into 1 g of each pure mineral (i.e., muscovite, biotite, and quartz) and reacted for 10 min at pH 3.5 ± 0.2. Then, the sample was filtered using a Whatman GF/C glass microfiber filter (1.2 μm pore size), dried at room temperature, and analyzed using the FT-IR with the aid of attenuated total reflectance to avoid further pretreatment. The transmittance was measured at a resolution of 4 cm^−1^ in a range of 4000–500 cm^−1^.

### 2.3. Bench Flotation Tests on the Soil Sample

#### 2.3.1. Optimization of the Soil Sample Mechanical Preparation

The soil sample (≤2 mm) was milled to determine the appropriate soil particle size for the froth-flotation separation processes. For the milling experiment, a device made by the laboratory was used (Figure S3). Forty percent of the jar with a capacity of 1.1 L was filled with rods, and its pore was filled with the soil slurry (1 mL/1 g = tap water/soil). Then, the slurry in the jar was ground at 60% of the rod mill’s critical rotation speed (i.e., 133 rpm). In addition, sieving was performed to identify the most proper soil particle size group for removing F from the soil using the froth-flotation separation processes. The soil sample was divided into particle size groups using appropriate sieves. Three different combinations of soil samples (i.e., milling all ≤2 mm soil sample, milling the 2–0.5 mm soil and mixing it with all ≤0.5 mm soil sample, and milling the 2–0.5 mm soil sample and mixing it with the 0.5–0.05 mm soil (i.e., discarding particles less than 0.05 mm)) were subjected to the froth-flotation separation processes to select the sieving-and-milling combination that exhibited the optimal F-removal efficiency. The froth-flotation separation experiment on the real target soil sample used a Sub-A flotation separator (KHD Humboldt Wedag AG, Cologne, Germany) with an air flow adjuster. The volume of the flotation separation cell was 1.5 L.

#### 2.3.2. Determination of Collector Dosage

At the slurry concentration of a solid (soil sample)–liquid (tap water) ratio of 1:39 (*w*/*w*), the removal efficiency of F was evaluated with varying concentrations of collector (Armac-T = 100, 200, 300, 400, and 500 mg/kg-soil) when applying the optimal milling-and-sieving combination for the target soil sample. The pH was adjusted to 3.5 ± 0.2, and 50 mg/kg-soil of the frother (AF65) was added and reacted for 2 min. The generated foams and floating product were recovered while injecting compressed air at a flow rate of 2 L/min for 2 min. The recovered floating product and the residue in the cell were filtered using a vacuum filter, and the filtering cakes were dried at 105 °C in an oven. The cakes were then weighed to calculate the flotation yield (Equation (1)). The F concentration in the cake was measured using the alkali fusion method. The F-removal efficiency was calculated using Equation (2) to determine optimal collector dosage:

(2) $$R_{f}=\frac{R\times r^{\prime }-S\times s^{\prime }}{R\times r^{\prime }}\times 100$$

where *R_f_* is the removal efficiency of F (%), *R* is the weight of the raw sample (kg), *r′* is the F concentration of the raw sample (mg/kg), *S* is the weight of the residue in the cell (kg), and *s′* is the F concentration of the residue in the cell (mg/kg).

#### 2.3.3. Multi-Stage Flotation Process

To remediate the soil sample enriched with F, the optimal conditions were applied in the first flotation process: a solid–liquid ratio of 1:9 (*w*/*w*), stirring rate of 1200 rpm, pH of 3.5 ± 0.2, Armac-T dosage of 400 mg/kg, AF65 dosage of 50 mg/kg, reaction time of 2 min, compressed air injection rate of 2 L/min, and foam recovery time of 2 min. In the second flotation process (a continuation of the process for the residue of the first flotation process), tap water was added to the residue of the first process to produce the slurry (1:9 *w*/*w*), and half of the amount of Armac-T used in the first flotation process was used. The other conditions remained the same. To examine the differences in particle sizes between floating product and residue in each flotation process, the particle sizes were analyzed.

## 3. Results and Discussion

### 3.1. Distribution of F Concentration in Soil

The weight composition and F concentration for each soil particle size group are listed in Table 1. The ≤0.5 mm soil particles [28], which are generally known to be suitable for flotation separation, accounted for 73.6% of the target soil. In the ≤0.075 mm soil particles, 1564.3 ± 159.5 mg/kg of F was measured, which was only 1.33 times the F concentration of the ≤2 mm soil group (1197.7 ± 105.0 mg/kg) (Table 1). In general, contaminants introduced into the soil from anthropogenic sources are likely to be adsorbed to the surfaces of particulates with a large specific surface area. Hence, as the soil particle size decreases, the concentration of contaminants increases [32,33]. In contrast, the concentrations of contaminants of natural origin, such as those in the target soil of this study that are derived from mineral weathering [19], are unlikely to show significant differences with soil particle size. Furthermore, by analyzing the F concentrations of the mica minerals recovered by hand-picking from the ≥ 2 mm gravel in the sample, 3020 ± 202 mg/kg of F was determined. This indicates that the accumulation of F in the soil was caused by the weathering of mica minerals [19].

### 3.2. Micro Flotation Tests

#### 3.2.1. pH Optimization

**Figure 1:**
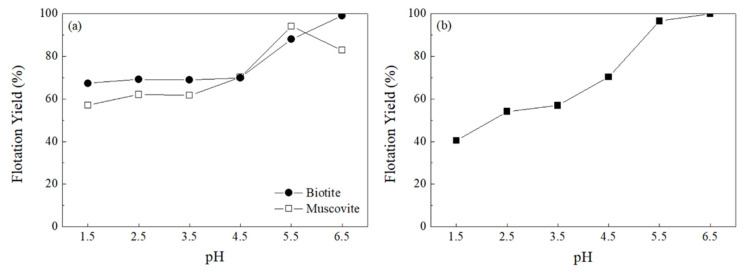
Flotation yield of (**a**) mica (muscovite and biotite) and (**b**) a mixture of biotite and quartz (1:1, *w*/*w*) with varying pH levels from 1.5 to 6.5.

To establish the optimal conditions for removing mica minerals that contain F from soils using the flotation separation technique, the flotation yields of the pure minerals (biotite and muscovite) were evaluated according to pH. As the pH increased, the flotation yields showed an increasing trend (Figure 1a). The Armac-T, the amine-based collector used in this study, is known to physically adsorb to mineral surfaces by electrostatic attraction [34]. Because mica minerals have an isoelectric point of pH ≤ 2 [34,35], the flotation yield likely increased because of the strengthened electrostatic attraction between Armac-T and mica minerals with increasing pH. The flotation yield according to pH for the mixed sample with biotite and quartz (as a major mineral component of soil) at 1:1 (*w*/*w*) was also evaluated. As the pH increased, the flotation yield showed an increasing trend (Figure 1b), but a high flotation yield does not necessarily reflect the effective separation of quartz and biotite. Because the quartz and biotite both floated at pH 5.5 and 6.5, it seems difficult to separate only F-containing mica minerals from the soil. At lower pH, the quartz and biotite were separated more effectively, when observed with the naked eye by color of floating product, but as the flotation yield decreased, the optimal pH condition was determined to be 3.5. This is because, at pH 3.5, not only can a separation of biotite and quartz similar to that at pH 2.5 be achieved, but the generation of acidic wastewater can also be minimized.

#### 3.2.2. Surface Characterization Using FT-IR

**Figure 2:**
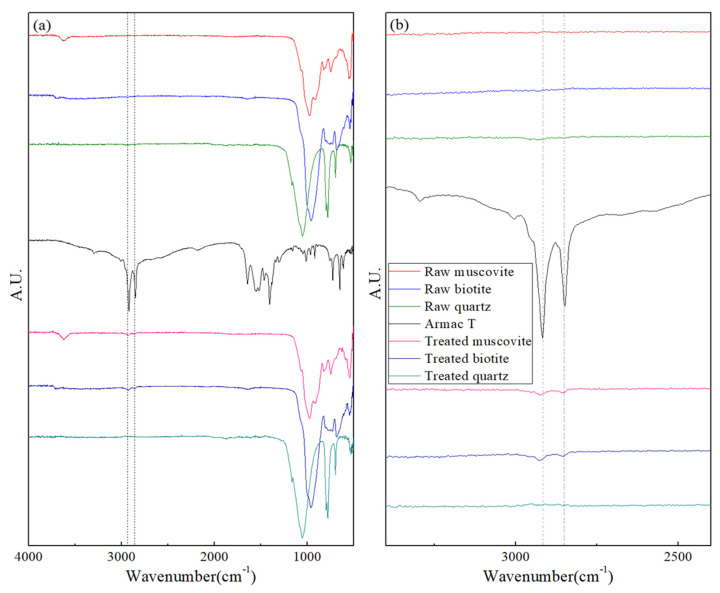
FT-IR spectra of 4000–500 cm^−1^ region of muscovite, biotite, and quartz before and after treatment with Armac-T at pH 3.5 ± 0.2 (**a**). The image on the right (**b**) is an enlargement of the 3400–2400 cm^−1^ region in the left image.

Figure 2 shows the results of the FT-IR analysis conducted to observe the reaction mechanisms of the collector (Armac-T) and mica minerals. The muscovite and biotite samples treated with Armac-T under pH conditions of 3.5 ± 0.2 showed significant –CH stretching vibration peaks (at 2918 and 2848 cm^−1^) [36,37], which were not observed in the control group (i.e., without the addition of Armac-T), and were strongly observed in Armac-T. However, in the quartz sample, these peaks were not observed regardless of Armac-T addition. This indicates that, at this pH, Armac-T adsorbs to mica minerals but does not adsorb to quartz, which is a major mineral component of the soil. Therefore, quartz and mica can be effectively separated using the proposed flotation separation processes and operating conditions.

#### 3.2.3. Particle Size Optimization

**Figure 3:**
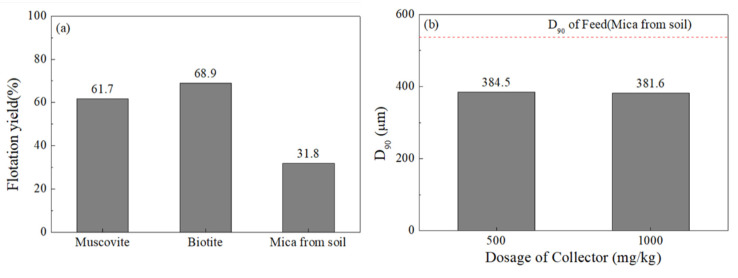
Flotation yield of muscovite, biotite, and mica collected manually from gravel (>2 mm) in the sample and milled (**a**), and D_90_ of the floating product according to collector dosage (Armac-T) (**b**).

The flotation yield at pH 3.5 was evaluated for the mica minerals hand-picked from the ≥2 mm gravel samples collected from the study site. Although, at pH 3.5, the muscovite pure mineral and the biotite pure mineral showed a flotation yield of 61.7% and 68.9%, respectively, the mica recovered from the field sample only showed a flotation yield of 31.8% (Figure 3a). The floating product was thus collected, and their particle sizes were analyzed. The 90% passing cumulative particle size (D_90_) was approximately 380 μm, regardless of the collector dosage (Figure 3b). This indicates that mica mineral particle sizes >380 μm are inappropriate for flotation separation (i.e., they precipitate regardless of the injected amount of collector). Therefore, it is necessary to mill the target soil to <380 μm particle sizes to remove F derived from mica minerals using the flotation separation technique.

### 3.3. Bench Flotation Tests

#### 3.3.1. Optimal Procedures for Soil Sample Mechanical Preparation

The target mineral cannot be separated unless the mixed and agglomerated minerals are separated first. The degree of liberation refers to the degree to which minerals are separated from one another and has a value of 100 if all minerals are completely separated [38,39]. The ideal degree of liberation for separating minerals is 100, but it is practically difficult and unnecessary to achieve this value. Moreover, if a sample is milled too much (to achieve a higher degree of liberation), a large number of fine particles are generated, and the reagent dosage increases. Moreover, it causes the entrapment of non-target minerals, thus lowering the separation efficiency [40]. In this regard, the appropriate milling of samples is indispensable for effective flotation separation. This study applied the flotation separation technique as a remediation technology to achieve the soil environmental standard, not the high-purity separation of minerals. Therefore, the soil samples were milled to maintain a simple remediation process with flotation rather than to achieve a high degree of liberation of minerals. After milling the ≤2 mm soil samples by a rod mill, which was fabricated in the laboratory for 10 min, a D_90_ value of 310 μm was achieved (Figure S4).

The F concentration in the floated soil, after milling the ≤2 mm soil samples for 10 min and applying the froth-flotation process, was 1064 mg/kg (Table S1). This is lower than the F concentration of 1198 mg/kg in the original soil sample (Table 1), and it demonstrated that the F-containing mica was not effectively separated from the soil. A large number of fine particles were generated by milling the entire sample, and it is presumed that the separation efficiency decreased [41,42]. Therefore, to reduce the generation of fine particles, the 0.5–2.0 mm particle size group was separated through sieving and milled for 10 min (Figure S5). It was then mixed with the ≤ 0.5 mm particle size group and subjected to the froth-flotation process. Subsequently, the F concentration in the floated soil was determined to be 1214 mg/kg (Table S1). Although this is a little higher than the F concentration in the original soil sample, it was still difficult to determine whether the F was concentrated in the floated soil. Therefore, the ≤0.05 mm particle size group was removed in advance, as these particles contain 1.3 times more F than the original soil (Table 1), and may lower the separation efficiency [41,42]. Then, the 0.5–2.0 mm particle size group was milled for 10 min (Figure S5) and mixed with the 0.05–0.5 mm particle size group before performing the froth-flotation process. The F concentration in the floated soil was 1981 mg/kg, indicating that effective separation of the F-containing mica from the soil was achieved (Table S1). Overall, a strategy to remediate the target F-enriched soil was established: separate and mill the 0.5–2.0 mm particle size group, mix it with the 0.05–0.5 mm particle size group, and subject the mixture to the froth-flotation process.

#### 3.3.2. Determination of Collector Dosage

**Figure 4:**
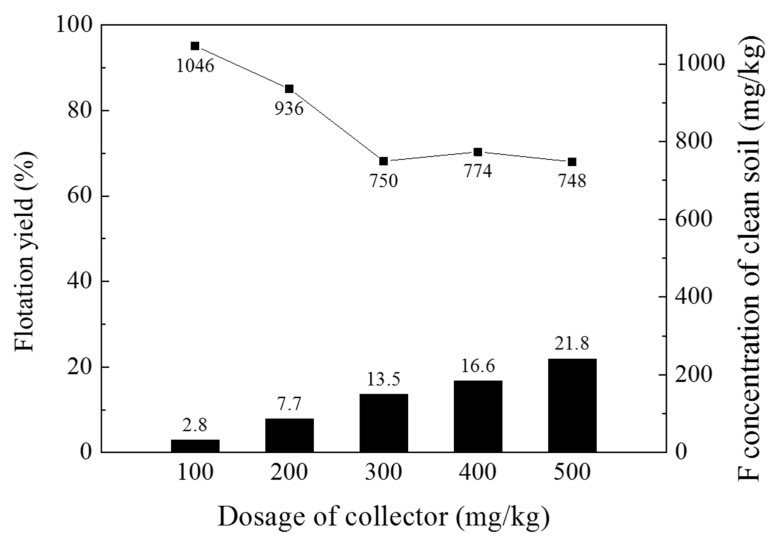
Flotation yield (left y-axis) and F concentration of clean soil (right y-axis) with varying dosages of the collector (Armac-T) from 100 to 500 mg/kg. The experimental conditions were as follows: a solid–liquid ratio of 1:39 (*w*/*w*), stirring rate of 1200 rpm, pH 3.5 ± 0.2, AF65 dosage of 50 mg/kg, reaction time of 2 min, compressed air injection rate of 2 L/min, and foam recovery time of 2 min.

After performing the optimum sample mechanical preparation (sieving-and-milling) strategy described in Section 3.3.1 for the target soil sample, a flotation separation experiment was conducted with different amounts of the collector. Figure 4 shows the flotation yield of the soil sample and the F concentration of the residual soil at various collector concentrations from 100–500 mg/kg. When the collector dosage was 300 mg/kg, 13.5% of the target sample floated. After the process, the F concentration of the residual soil was 750 mg/kg. When the collector dosages were 400 and 500 mg/kg, the F concentrations of the residual soils were 774 and 748 mg/kg, respectively, similar to the 300 mg/kg collector result. However, 16.6 and 21.8% of the target samples floated, and the amount of soil that had to be treated as waste increased. Overall, the optimal collector dosage for the froth-flotation separation process was determined to be 400 mg/kg, with consideration of the reduction of the amount of soil that needs to be removed as waste and the error that could occur in the field process.

#### 3.3.3. Application of Multi-Stage Flotation Processes

Based on the determined sample mechanical preparation strategy (i.e., sieving-and-milling condition) and collector dosage, the froth-flotation process was applied to the target soil sample. The results showed that the target soil flotation yield was 24% and the F concentration of the residual soil was 715 mg/kg (Table 2). Because the Korean worrisome level of soil contamination (level 2 area: 400 mg/kg) was not met, the second froth-flotation process was applied.

For the second flotation process, the collector dosage decreased by half [43], and all other conditions remained the same. In the second flotation process (using the residue in the first flotation), the flotation yield was 7% and the F concentration in the residual soil was 637 mg/kg (Table 2). An analysis of the particle sizes of the floating product and the residue in the first and second flotation processes (Table S2) showed that soil particles ≥ 350 μm did not float. This coincides with the optimal particle size of the froth-flotation process derived in this study. The first flotation process removed (by floating) soil particles ≤ 350 μm. This means that even if the second flotation process is performed with a reduced ratio of this particle size group, effective removal of the remaining F-enriched soil particles does not occur.

Therefore, after finishing the first flotation process and before performing the second, the residue of the first flotation process was subjected to additional milling. Based on the preliminary milling test for < 0.5 mm soil (Figure S6), milling for 3 min produced particles with a D_90_ of 279 μm (Table S2). When these particles were subjected to the second flotation process, a flotation yield of 28.3% and F concentration of 355 mg/kg in the residual soil were obtained. This is because the F-enriched soil particles (i.e., mica minerals) included in the particle size group that could not float were milled down to a particle size that could float.

**Figure 5:**
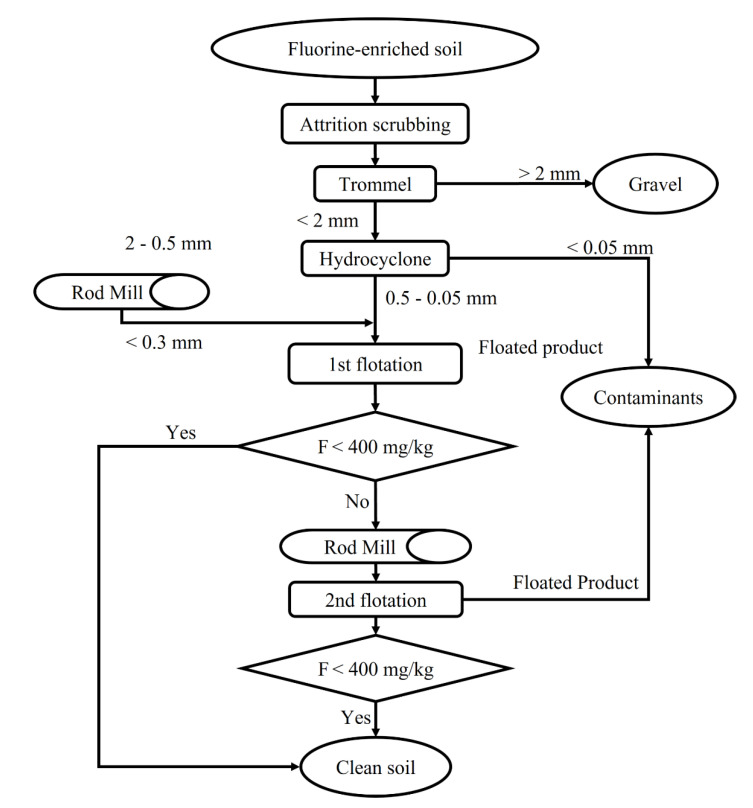
Remediation strategy for F-enriched soil derived from mica using the proposed froth-flotation separation process.

The overall remediation process for mica-derived F-enriched soil using the flotation separation technique is shown in Figure 5. The target soil used in this study showed a relatively high F concentration of 1198 mg/kg. To satisfy the Korean worrisome level of soil contamination of 400 mg/kg, relatively large volumes of soil particles had to be removed as the floating product. However, it is meaningful that this is the only way that can remove (reduce the concentration of) F that is strongly chemically bonded, such as that in F-enriched soil of natural origin (including mica). In addition, if the first flotation process achieves the remediation goal depending on site conditions, the amount of discarded soil would be substantially smaller.

## 4. Conclusions

The froth-flotation separation technique was applied to treat F-enriched soils of natural origin that were derived from mica minerals. According to previous sequential extraction analysis results, 99% of the F in the target soil is in the residual fraction [19], and therefore, remediation options such as chemical soil washing and electrokinetics cannot be applied. The optimal conditions for the froth-flotation separation process proposed in this study were as follows. The target soil and tap water were mixed at a ratio of 1:9 (*w*/*w*), and the pH titrated to 3.5 ± 0.2 using H_2_SO_4_. Then, the samples were reacted at a stirring rate of 1200 rpm for 2 min with a collector (Armac-T) and frother (AF 65), respectively. The collector dosage for the first flotation process was 400 mg/kg, and that for the second flotation process was 200 mg/kg. A sample mechanical preparation strategy for the soil sample to be introduced in the first flotation process involved discarding <0.05 mm particles, milling those in the range of 0.5–2.0 mm until < approx. 0.3 mm, and mixing 0.05–0.5 mm particles. If the Korean worrisome level of soil contamination of 400 mg/kg is not satisfied in the first flotation process, the second flotation process can be applied with milling of the residue (< about 0.3 mm) of the first flotation process. When F originates from minerals, such as in the target soil, it is difficult to remove it using acid washing or electrokinetic methods. In such cases, the froth-flotation separation method verified in this study can be used as an important alternative remediation technique.

## Figures and Tables

**Table 1:** Particle size distribution and total F concentrations of the soil sample.

Particle Size (mm)	Weight Composition (%)	Total F Concentration (mg/kg)
<2	100	1197.7 ± 105.0
0.5–2.0	26.4 ± 1.6	1126.3 ± 272.4
0.15–0.5	28.0 ± 1.3	1036.3 ± 34.0
0.075–0.15	8.4 ± 0.5	1564.3 ± 159.5
<0.075	37.2 ± 1.9	1594.3 ± 42.3

Note: The symbol “±” denotes the standard deviation (*n* = 3).

**Table 2:** Flotation yield and F concentration in treated soil (residue) with repeated application of the proposed froth-flotation separation process.

Sample Classification	Flotation Yield (%)	F Concentration in Treated Soil (mg/kg)
After performing first flotation process	24	715
After performing second flotation process without additional milling	7	637
After performing second flotation process with additional milling to residue of first flotation process	28.3	355

## Data Availability

Data are contained within the article.

## Supplementary Materials

The following supporting information can be downloaded at: https://www.mdpi.com/article/10.3390/ijerph19031775/s1, Figure S1: XRD peaks of pure minerals, Figure S2: Hand-picked mica from sample (>2 mm), Figure S3: Specifications of Jar and Rod (Jar inner diameter: 100 mm, Jar volume: 1,100 mL, Jar and rod material: SU304, Rod: diameter 8 mm, length 140 mm, density 7.94 g/cm^3^), Figure S4: D90 of soil sample (Under 2 mm) plotted against the grinding time, Figure S5: D90 of soil sample (0.5 to 2 mm) plotted against the grinding time, Figure S6: D90 of soil sample (Under 0.5 mm) plotted against the grinding time, Table S1: Froth-flotation separation results under various milling conditions, Table S2: Particle size distribution of floating product and residue of the 1st and 2nd froth-flotation processes with and without milling prior to the 2nd flotation.
