# Supplementary material for: Froth-Flotation Separation as an Alternative for the Treatment of Soil Enriched with Fluorine Derived from Mica
Source: Int J Environ Res Public Health. 2022 Feb 4;19(3):1775. doi: 10.3390/ijerph19031775 (PMC8834934; doi:10.3390/ijerph19031775)
Supplement: Supplementary file 1 [file ijerph-19-01775-s001.zip › ijerph-1496617-supplementary.pdf]

Supplementary Data Cover Sheet

Title: Froth-flotation separation as an alternative for the treatment of soil enriched with fluorine derived from mica

Authors: Jeonghwan Cho[a], Moon Young Jung[b], Hwan Lee[c], Jinsung An[a,b,*]

[a]*Department of Environment Safety System Engineering, Semyung University, 65 Semyung-ro, Jecheon-si, Chungcheongbuk-do 27136, Republic of Korea*

[b]*Department of Biological & Environmental Engineering, Semyung University, 65 Semyung-ro, Jecheon-si, Chungcheongbuk-do 27136, Republic of Korea*

[c]*SG Institute of Environment Science & Technology, 42, Soryong 1-gil, Gunsan-si, Jeollabuk-do, Republic of Korea*

[*]Correspondence: E-mail address: jsan@semyung.ac.kr, Phone: +82-43-649-1335, Fax: +82-43-649-1779.

Submitted to *International Journal of Environmental Research and Public Health*

Prepared on November 21th, 2021

Number of Pages: 9
Number of Figure: 5
Number of Table: 2

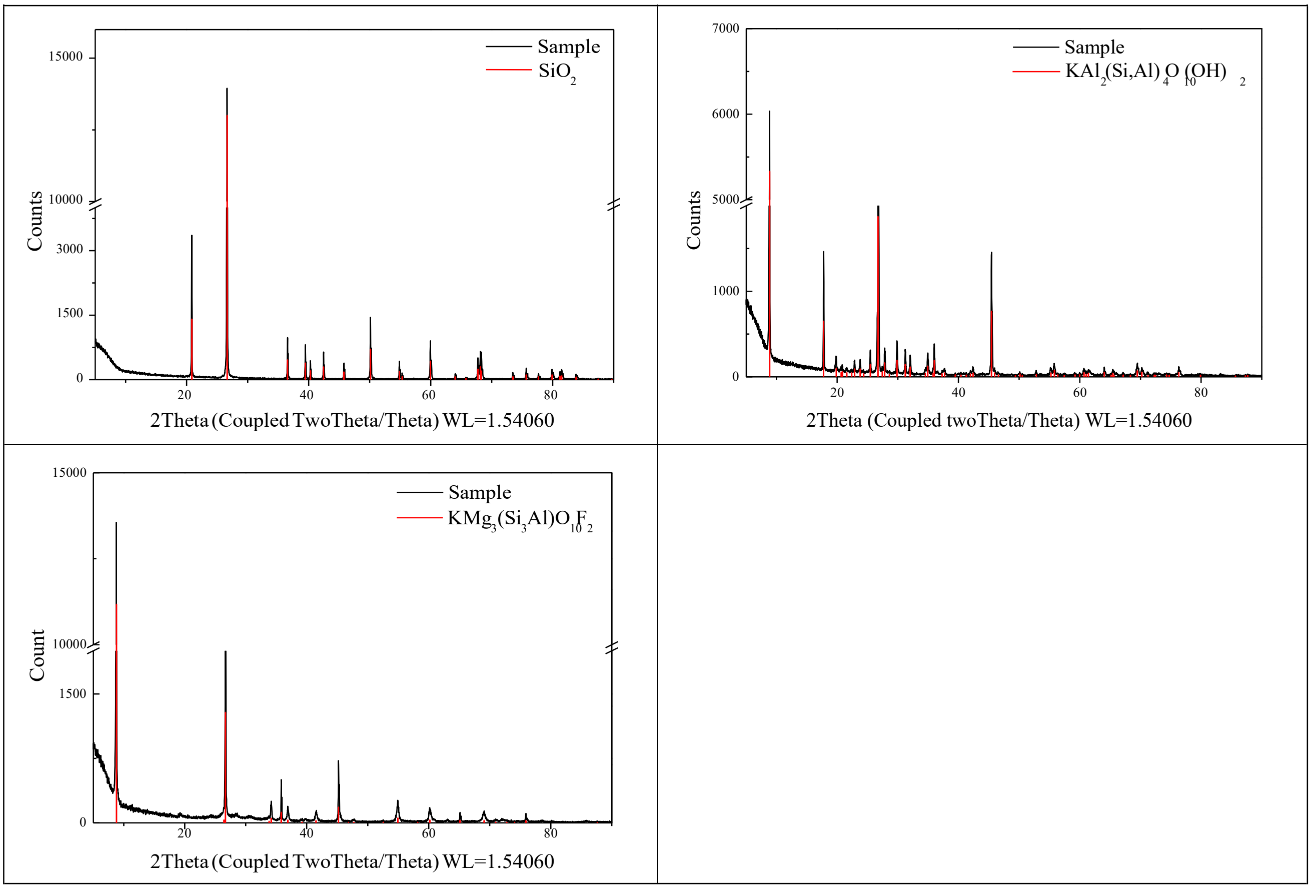

Figure S1. XRD peaks of pure minerals

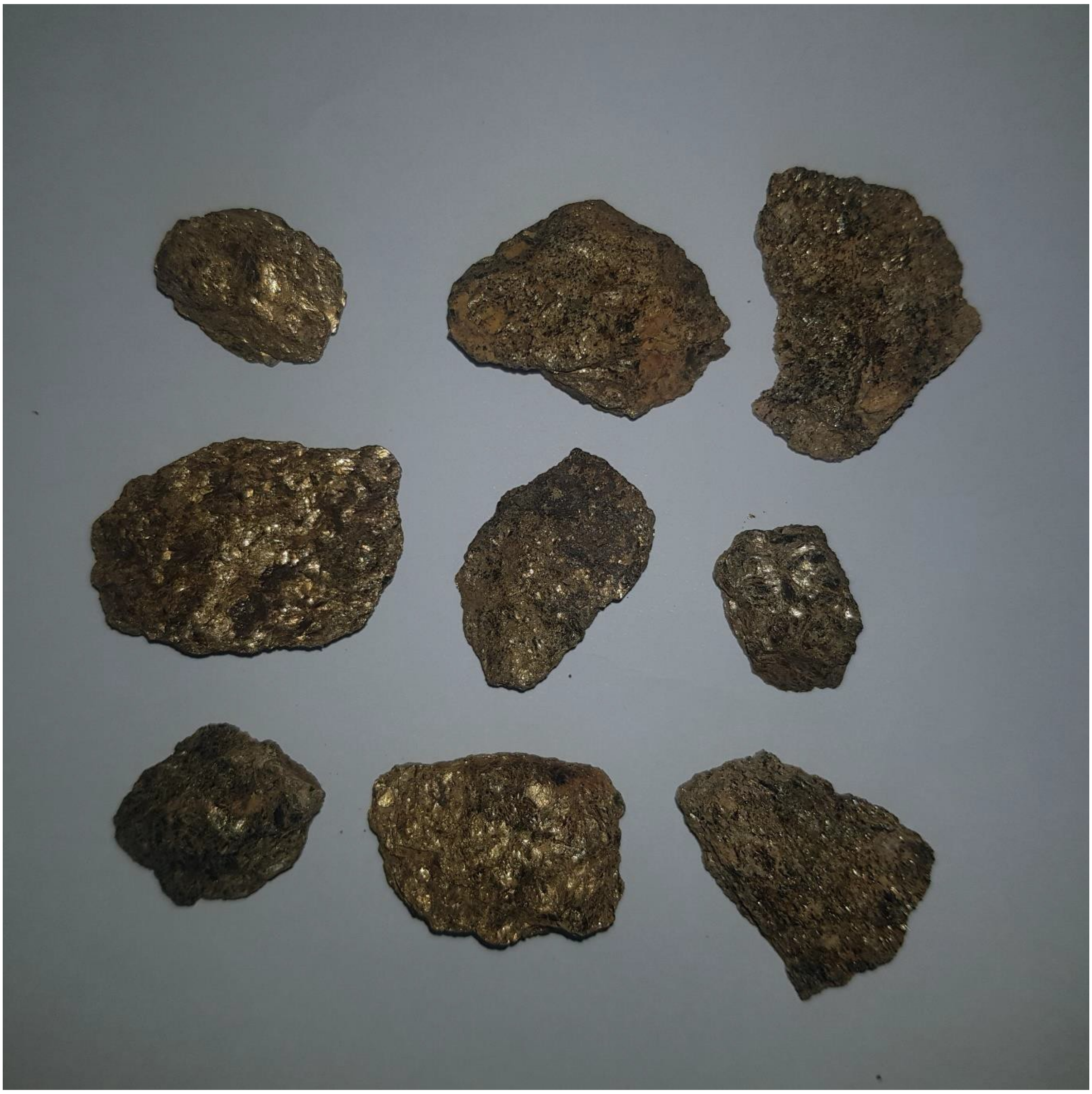

Figure S2. Hand-picked mica from sample (> 2 mm)

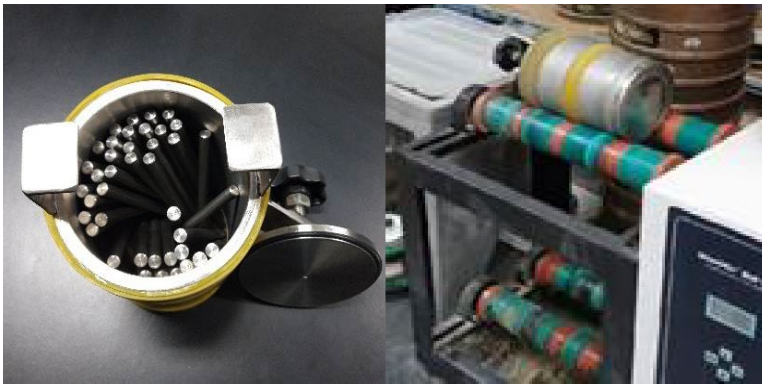

Figure S3. Specifications of Jar and Rod (Jar inner diameter: 100 mm, Jar volume: 1,100 mL, Jar and rod material: SU304, Rod: diameter 8 mm, length 140 mm, density 7.94 g/cm^3).

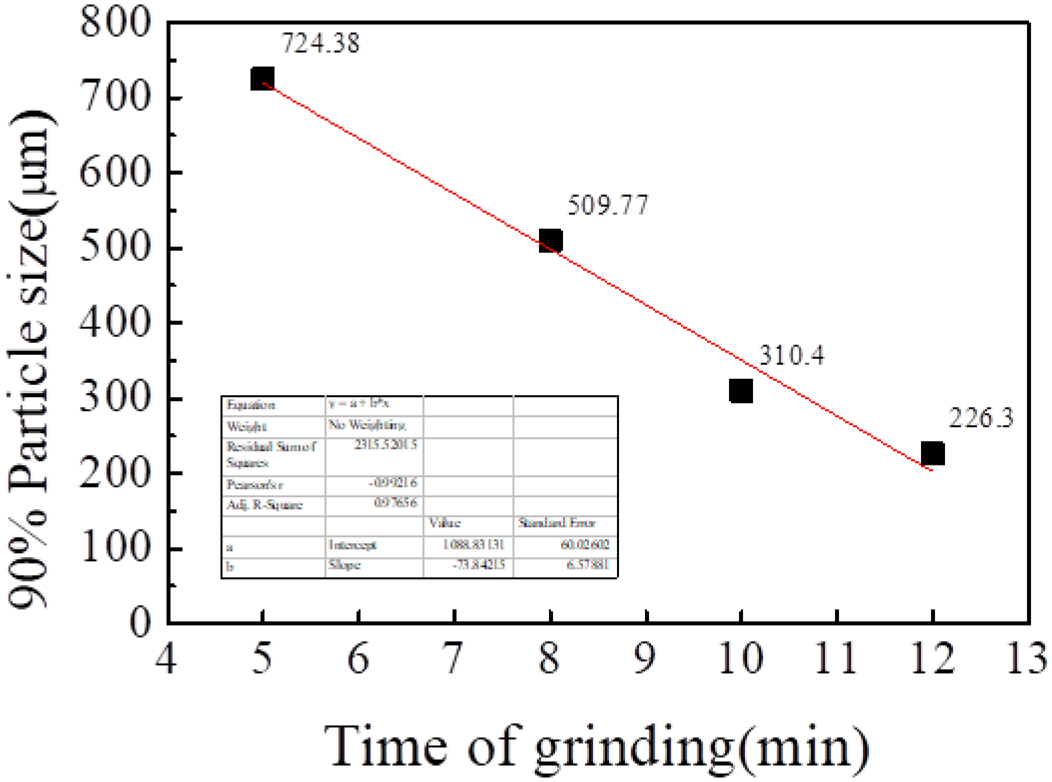

Figure S4. D90 of soil sample (Under 2 mm) plotted against the grinding time

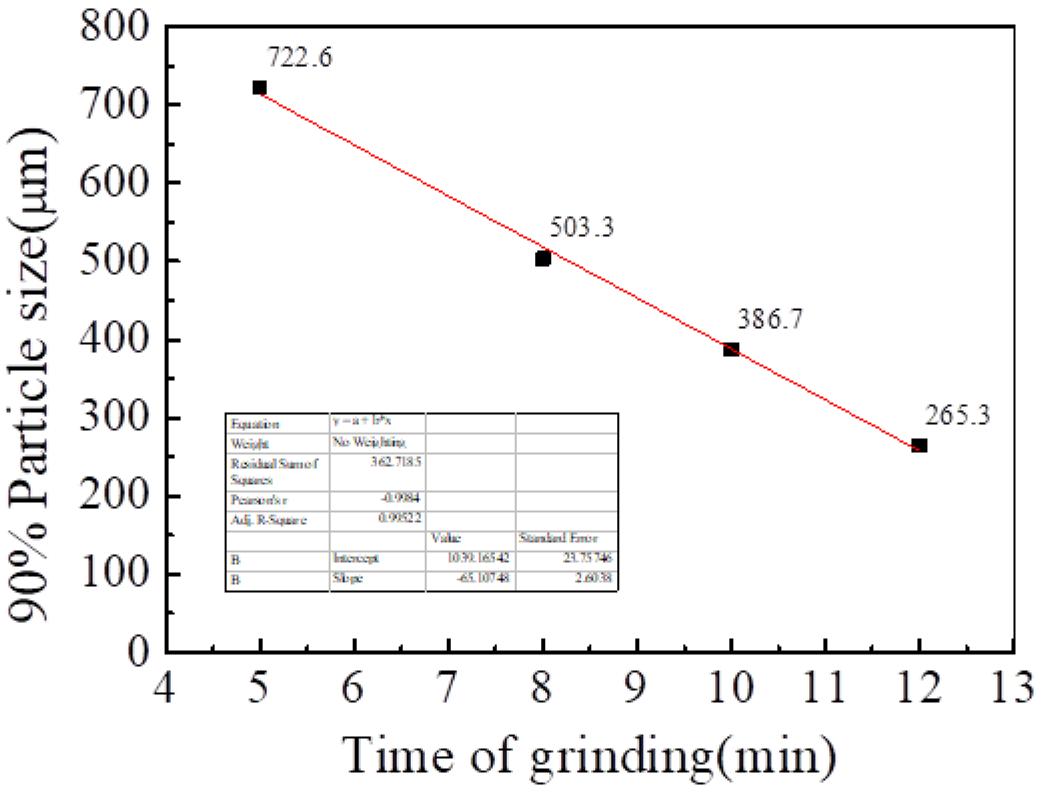

Figure S5. D90 of soil sample (0.5 to 2 mm) plotted against the grinding time

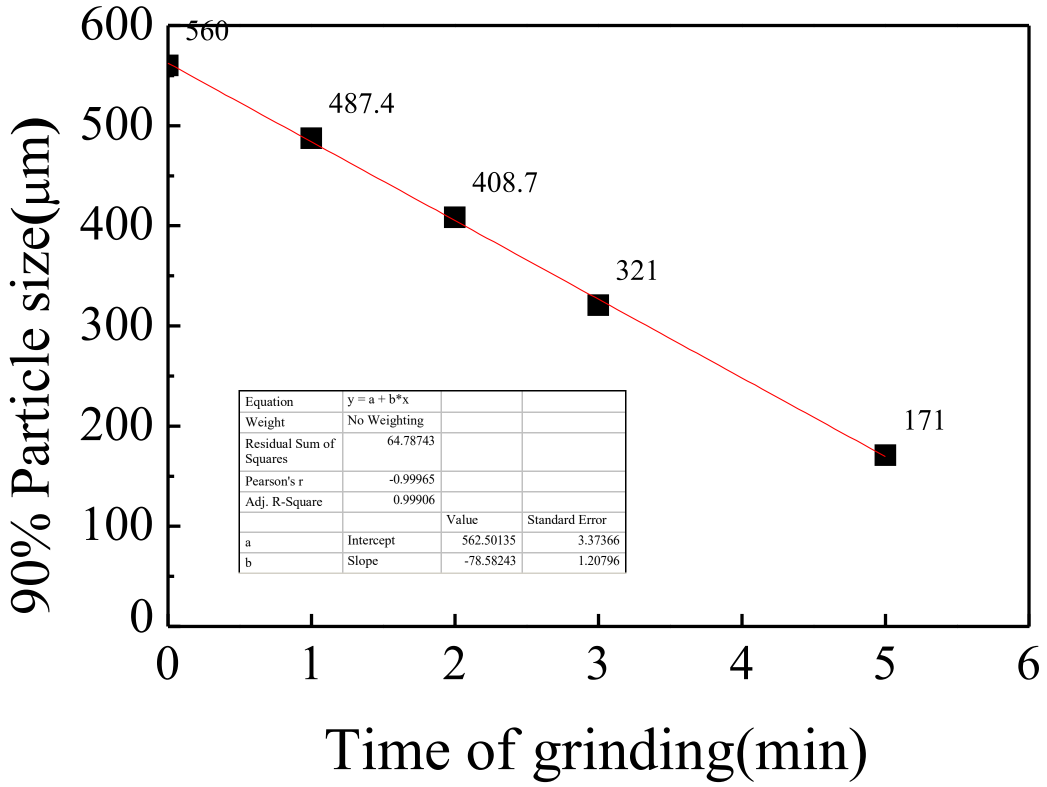

Figure S6. D90 of soil sample (Under 0.5 mm) plotted against the grinding time

Table S1. Froth-flotation separation results under various milling conditions

Milling condition	F concentration of enriched soil (mg/kg)	Flotation yield (%)
Milling ≤2 mm soil for 10 min	1064	19.5
Milling 0.5–2.0 mm soil for 10 min and mix with ≤0.5 mm soil	1214	11.3
Milling 0.5–2.0 mm soil for 10 min and mix with 0.05–0.5 mm soil	1981	23.0

Table S2. Particle size distribution of floating product and residue of the 1st and 2nd froth-flotation processes with and without milling prior to the 2nd flotation

	Feed	Floating product	Residue
1st flotation	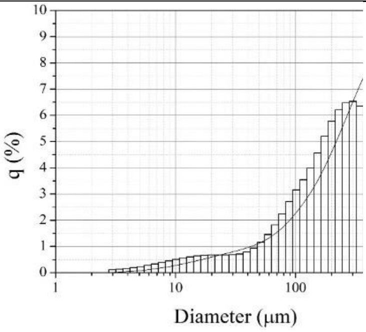	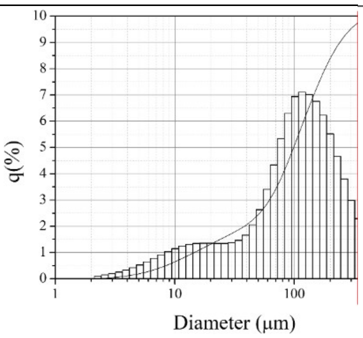	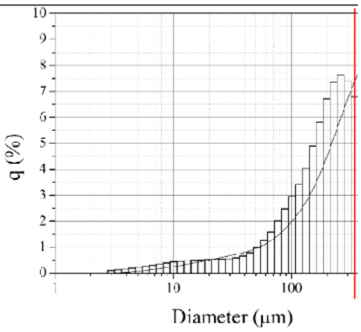
2nd flotation without milling	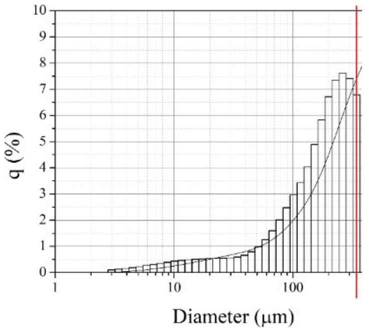	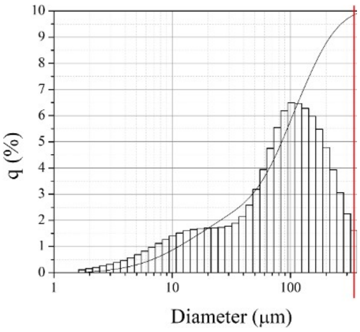	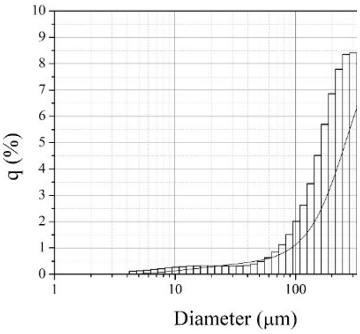
2nd flotation with milling	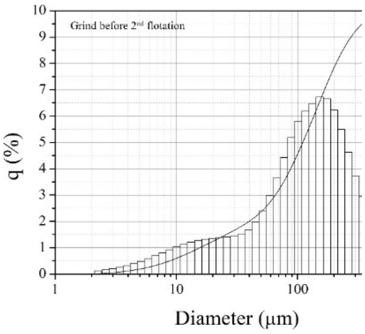	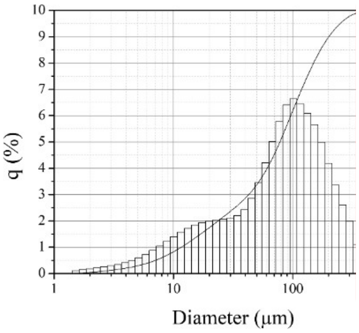	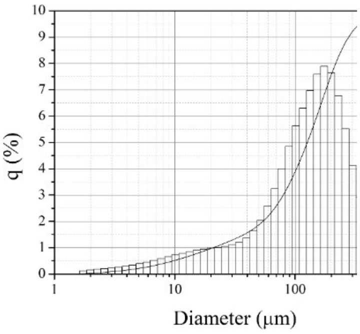

1st flotation feed: D_{10} = 43 μm, D_{90} = 552 μm; 1st flotation floating product: D_{10} 15 μm, D_{90} 241 μm; 1st flotation residue: D_{10} 73 μm, D_{90} 521 μm; 2nd flotation feed without milling: D_{10} 73 μm, D_{90} 521 μm; 2nd flotation floating product without milling: D_{10} 11 μm, D_{90} 216 μm; 2nd flotation residue without milling: D_{10} 92 μm, D_{90} 574 μm; 2nd flotation feed with milling: D_{10} 16 μm, D_{90} 276 μm; 2nd flotation floating product with milling: D_{10} 12 μm, D_{90} 200 μm; 2nd flotation residue with milling: D_{10} 21 μm, D_{90} 279 μm
